# In Vivo Tumor-Targeted Dual-Modality PET/Optical Imaging with a Yolk/Shell-Structured Silica Nanosystem

**DOI:** 10.1007/s40820-018-0216-2

**Published:** 2018-07-16

**Authors:** Sixiang Shi, Feng Chen, Shreya Goel, Stephen A. Graves, Haiming Luo, Charles P. Theuer, Jonathan W. Engle, Weibo Cai

**Affiliations:** 1 Department of Materials Science and Engineering, University of Wisconsin-Madison, Madison, WI USA; 2 Department of Radiology, University of Wisconsin-Madison, Madison, WI 53705-2275 USA; 3 Department of Medical Physics, University of Wisconsin-Madison, Madison, WI USA; 4 TRACON Pharmaceuticals, Inc., San Diego, CA USA; 5 University of Wisconsin Carbone Cancer Center, Madison, WI USA

**Keywords:** Hollow mesoporous silica nanoparticle (HMSN), Quantum dot (QD), Molecular imaging, Positron emission tomography (PET), Optical imaging, CD105/endoglin

## Abstract

Silica nanoparticles have been one of the most promising nanosystems for biomedical applications due to their facile surface chemistry and non-toxic nature. However, it is still challenging to effectively deliver them into tumor sites and noninvasively visualize their in vivo biodistribution with excellent sensitivity and accuracy for effective cancer diagnosis. In this study, we design a yolk/shell-structured silica nanosystem ^64^Cu-NOTA-QD@HMSN-PEG-TRC105, which can be employed for tumor vasculature targeting and dual-modality PET/optical imaging, leading to superior targeting specificity, excellent imaging capability and more reliable diagnostic outcomes. By combining vasculature targeting, pH-sensitive drug delivery, and dual-modality imaging into a single platform, as-designed yolk/shell-structured silica nanosystems may be employed for the future image-guided tumor-targeted drug delivery, to further enable cancer theranostics.
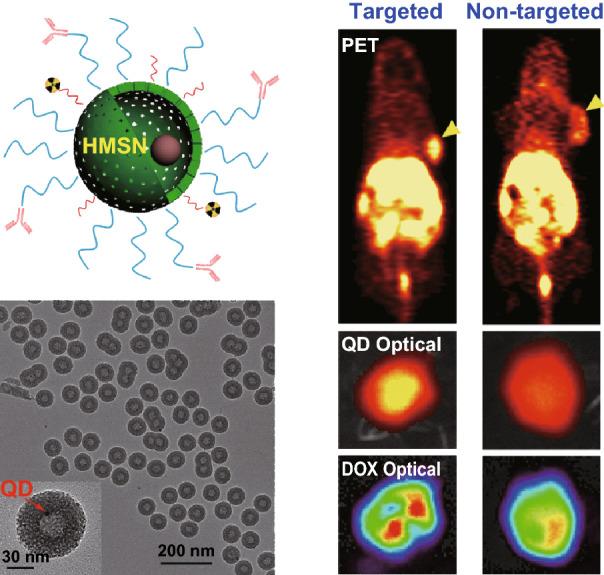

## Highlights

- A hybrid yolk/shell nanosystem was generated with quantum dot as the core and hollow mesoporous silica as the shell.
- Dual-modality PET/optical imaging was conducted to achieve synergistic cancer diagnosis that combines the advantages of both PET and optical imaging.
- Successful tumor vasculature targeting was achieved, which significantly enhanced tumor retention and targeting specificity.

## Introduction

In the last decade, rapid development of nanotechnology has dramatically stimulated the progress in design and synthesis of multifunctional nanosystems that can potentially be used for cancer-targeted imaging and therapy [1–5]. Among them, silica nanoparticles (NPs) have been one of the most widely studied nanosystems, due to their facile surface chemistry and non-toxic nature [6–8]. Silica-based NPs are “generally recognized as safe” by the United States Food and Drug Administration (FDA), highlighting their great clinical relevance [6–8].

Hollow mesoporous silica NPs (HMSNs), which possess numerous nanoscale pores and a large cavity inside the silica shell, have been recently developed for drug delivery due to their low mass density, large surface area, high pore volume, and uniform and tunable pore size [8–10]. Tumor active targeting has been applied to HMSNs in vivo to significantly enhance the tumor uptake and reduce possible side effects, leading to a desirable nanoplatform for cancer treatment [11–13]. Positron emission tomography (PET) has been accomplished with radiolabeled HMSNs to invasively understand their biodistribution in living animals [11–13]. Compared to traditional nanosystems, radiolabeled actively targeted HMSNs combine cancer diagnosis and therapy into one single platform, making cancer theranostics possible.

However, one imaging modality is not enough to accurately represent the in vivo fate of HMSNs [14–16]. Although PET imaging is highly quantitative and sensitive and has unlimited tissue penetration, it can only depict the in vivo biodistribution of radioisotopes, whether they are attached to or free from the carrier NPs [17–20]. Therefore, PET imaging alone may possibly lead to a totally different readout, owing to different biodistribution profiles of NP agents and free radioisotopes, which results in a false diagnosis. To overcome this drawback, we have designed a quantum dot (QD)/HMSN yolk/shell nanosystem (QD@HMSN) that makes use of the optical properties of QDs for fluorescence imaging. Compared to other fluorescent emitters such as organic dyes, QDs have wider excitation and narrower emission spectra, higher quantum yields and minimal photo bleaching, and therefore can accurately render the biodistribution of HMSNs, providing synergistic diagnostic information in addition to PET [21]. The incorporated QDs were placed in the cavity and protected by the silica shells, which will not alter the intrinsic pharmacokinetics of HMSNs in the living systems.

Unlike small molecules or antibody, NPs have a relatively large size and exhibit suboptimal extravasation from vessels, which limits their applications in tumor cell targeting. Tumor vasculature targeting avoids the need of extravasation and has become one of the most effective active targeting methods for NPs [22]. Endoglin receptor (CD105) is a type I membrane glycoprotein, which highly proliferates in tumor neovasculature and plays an important role in tumor angiogenesis, growth and metastasis [23–26]. In this study, a chimeric human/murine anti-CD105 antibody, TRC105, was conjugated onto the surface of QD@HMSN yolk/shell nanosystems for effective tumor vasculature targeting and enhanced tumor diagnosis.

## Materials and Methods

### Materials

Chelex 100 resin (50–100 mesh), tetraethyl orthosilicate (TEOS), triethanolamine (TEA), (3-aminopropyl)triethoxysilane (APTES), and cetyltrimethylammonium chloride solution (CTAC, 25 wt%) were purchased from Sigma-Aldrich (St. Louis, MO). TRC105 was provided by TRACON Pharmaceuticals Inc. (San Diego, CA). Complete mouse serum was purchased from Jackson Immuno Research Laboratories (West Grove, PA). 1,4,7-Triazacyclononane-1,4,7-triacetic acid (NOTA) was purchased from Macrocyclics, Inc. (Dallas, TX). ^64^Cu was produced by a GE PETtrace cyclotron using the ^64^Ni(p,n)^64^Cu reaction. Water and all buffers were of Millipore grade and pre-treated with Chelex 100 resin to ensure that the aqueous solution was free of heavy metal. All other chemicals and buffers were obtained from Thermo Fisher Scientific (Fair Lawn, NJ).

### Synthesis of QD@HMSN Yolk/Shell Nanosystems

QD NPs (Qdot™ 705 ITK™ carboxyl quantum dots, Thermo Fisher Scientific, Fair Lawn, NJ) were first coated with dense silica (dSiO_2_) layer, by oil-in-water reverse micro-emulsion silica coating approach. In brief, Igepal CO-520 (NP-5, 1 mL) was dispersed in cyclohexane (20 mL) in a 100-mL three-necked flask and stirred at a slow rate for 5 min. Qdot705 (ITK organic quantum dots, 400 pmol) was added into CO-520 solution and stirred slowly for 2 h. Ammonia solution (0.14 mL, 30%) was then added into the mixture and stirred for another 2 h. TEOS was subsequently pumped into the reaction (100 μL h^−1^) for 2 h and stirred at room temperature for 40 h. When the reaction was completed, 1 mL methanol and 2 mL ethanol were added into the solution to precipitate the sample. The QD@dSiO_2_ samples were collected by centrifugation and washed with ethanol for three times.

As-synthesized QD@dSiO_2_ NPs were further coated with mesoporous silica NPs (MSNs) to form the mesoporous silica shell. In brief, 20 μg TEA and 10 mL CTAC (25 wt%) were dissolved into 20 mL water and stirred for 1.5 h. Ten milliliters of QD@dSiO_2_ solution (containing 200 pmol QDs) was then added and stirred for another 1.5 h. TEOS was subsequently added at the rate of 20 μL min^−1^ for 5 min. The reaction took place at 80 °C for 1 h. After the reaction was finished, QD@dSiO_2_@MSN solution was mixed with 636 mg Na_2_CO_3_ and moved to 50 °C water bath for 45 min to etch out dSiO_2_. The resulting QD@HMSN NPs were collected by centrifugation and washed with water for three times. To remove CTAC, the product was extracted for 24 h with a 1 wt% solution of NaCl in methanol at room temperature. This process was carried out for at least three times to ensure the complete removal of CTAC.

### Surface Engineering of QD@HMSN Yolk/Shell Nanosystems

NH_2_ groups were introduced onto the surface of QD@HMSN NPs for further surface engineering. QD@HMSN NPs were dispersed in 20 mL of absolute ethanol, followed by the addition of 1 mL of APTES. The system was sealed and kept at 86–90 °C for reaction for 24 h. When the reaction was completed, the NH_2_-modified QD@HMSN (QD@HMSN-NH_2_) was collected by centrifugation and washed with absolute ethanol for three times to remove the remaining APTES.

**Fig. 1:**
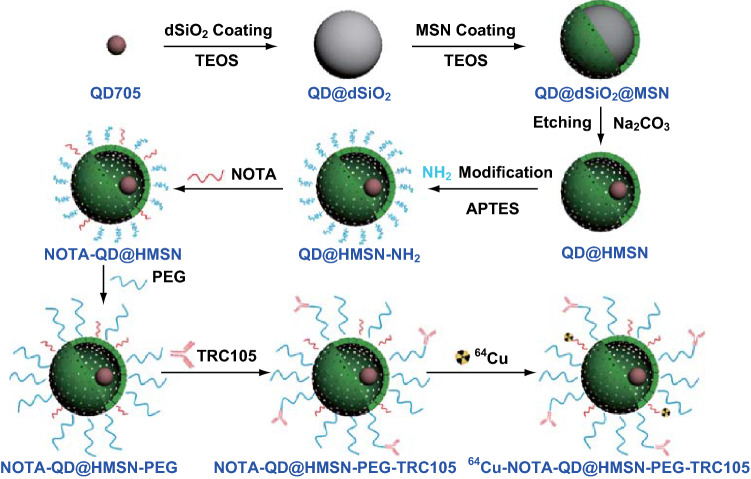
A schematic illustration of the synthesis and functionalization of QD@HMSN yolk/shell-structured silica nanosystem

For chelating radioisotopes, p-SCN-Bn-NOTA was conjugated onto QD@HMSN NPs based on the reaction between SCN and NH_2_ groups at pH 9 for 2 h (QD/NOTA molar ratio 1:500; of note, since QD is the seed of HMSN, the molar concentrations of QD and HMSN NPs were nearly identical). Fluorescein isothiocyanate (FITC) was conjugated onto QD@HMSN NPs with the same method for in vitro experiments. The resulting NOTA-QD@HMSN or FITC@HMSN was then conjugated with SCM-PEG_5k_-Mal based on the reaction between SCM group (succinimidyl carboxyl methyl ester) and remaining NH_2_ groups at pH 7 for 2 h (QD/PEG molar ratio 1:10,000). Meanwhile, TRC105 (QD/TRC105 molar ratio 1:20) was reacted with Traut’s reagent (TRC105/Traut’s reagent molar ratio 1:20) at pH 8 for 1 h to introduce free SH groups on the antibodies. Finally, as-prepared NOTA-QD@HMSN-PEG or FITC-QD@HMSN-PEG was mixed with TRC105-SH and reacted at pH 7.4 for 2 h in the presence of tris(2-carboxyethyl)phosphine (TCEP, to prevent oxidation of the thiol), generating the final products NOTA-QD@HMSN-PEG-TRC105 or FITC-QD@HMSN-PEG-TRC105 (Fig. 1).

### Cell Lines and Animal Models

All animal studies were conducted under a protocol approved by the University of Wisconsin Institutional Animal Care and Use Committee. 4T1 murine breast cancer cells were obtained from American Type Culture Collection (ATCC, Manassas, VA) and cultured according to the supplier’s instructions. When they reached ~ 80% confluence, the cells were harvested for tumor implantation. Four-to-five-week-old female Balb/c mice (Envigo, Indianapolis, IN) were each subcutaneously injected with 2 × 10^6^ 4T1 cells in the flank to generate the 4T1 breast cancer model. The mice were used for in vivo experiments when the tumor diameter reached 6–8 mm.

### In Vitro CD105 Targeting

Human umbilical vein endothelial cells (HUVECs, CD105 positive) were harvested and suspended in cold PBS with 2% bovine serum albumin at a concentration of 5 × 10^6^ cells mL^−1^, incubated with FITC-QD@HMSN-PEG or FITC-QD@HMSN-PEG-TRC105 at a concentration of 10 pmol mL^−1^ (based on QD concentration) for 30 min at room temperature. A blocking experiment (500 μg mL^−1^ of TRC105 added to the cells 3 h before administration of NPs) was also carried out to further validate the targeting specificity. Afterward, the cells were collected by centrifugation at 1000 rpm for 5 min and washed three times with cold PBS. The cells were analyzed using a BD FACSCalibur 4-color analysis cytometer equipped with 488 and 633 nm lasers (Becton-Dickinson, San Jose, CA) and FlowJo analysis software (Tree Star, Inc., Ashland, OR).

### Radiolabeling and In Vivo PET Imaging

^64^CuCl_2_ (74 MBq) was diluted in 300 µL of 0.1 M sodium acetate buffer (pH 5.5) and mixed with NOTA-QD@HMSN-PEG and NOTA-QD@HMSN-PEG-TRC105. The reactions were conducted at 37 °C for 45 min with constant shaking. The resulting ^64^Cu-NOTA-QD@HMSN-PEG and ^64^Cu-NOTA-QD@HMSN-PEG-TRC105 were purified by PD-10 size exclusion column chromatography using PBS as the mobile phase. NOTA-mediated ^64^Cu labeling proved to be stable in accordance with our previous studies.

^64^Cu-NOTA-QD@HMSN-PEG and ^64^Cu-NOTA-QD@HMSN-PEG-TRC105 were intravenously injected to 4T1 tumor-bearing mice. Serial PET scans were performed using a microPET/microCT Inveon rodent model scanner (Siemens Medical Solutions, USA, Inc.) at different time points (0.5, 3, 6, and 24 h) post-injection (p.i.). Quantitative data of ROI analysis on tumor and other organs were presented as percentage injected dose per gram of tissue (%ID/g). After the last scan at 24 h p.i., mice were killed under anesthesia for ex vivo biodistribution studies. Tumor, blood, and major organs/tissues were collected and weighted. The radioactivity in the tissue was measured using a γ counter (PerkinElmer) and presented as %ID/g (mean ± SD).

### Drug Loading and Ex Vivo Fluorescence Imaging

Doxorubicin (DOX) was loaded into QD@HMSN yolk/shell nanosystems as the model drug. In brief, DOX was dissolved in DMSO (10 mg mL^−1^) and added into NOTA-QD@HMSN-PEG or NOTA-QD@HMSN-PEG-TRC105 solutions (NP/DOX weight ratio 1:2; NP weight was measured after freeze-drying of QD@HMSN-PEG) for overnight incubation under room temperature. The DOX-loaded NOTA-QD@HMSN-PEG or NOTA-QD@HMSN-PEG-TRC105 (NOTA-QD@HMSN(DOX)-PEG or NOTA-QD@HMSN(DOX)-PEG-TRC105) was collected with centrifugation and washed with PBS for three times. The amount of DOX which was washed away was measured and calculated by UV–Vis absorbance at 480 nm, based on the standard equation [washed DOX weight = (UV–Vis intensity-0.0356)/0.00403] which was generated from the measurement of the DOX samples with known concentrations. The final loading capacity was calculated by the equation [loading capacity = (mixed DOX weight–washed DOX weight)/NP weight].

The drug release tests were performed by incubating DOX-loaded QD@HMSN yolk/shell nanosystems in both normal physiological environment (pH 7.4) and acidic environment (pH ~ 5). The DOX-loaded samples were centrifuged at 3, 6, 24, and 48 h post-incubation, and the supernatants were collected and measured by UV–Vis absorbance at 480 nm. The amount of the released DOX was calculated by the equation [released DOX weight = (UV–Vis intensity-0.0356)/0.00403]. The drug release rate was calculated by the equation [drug release (%) = released DOX weight/loaded DOX weight × 100%].

NOTA-QD@HMSN(DOX)-PEG and NOTA-QD@HMSN(DOX)-PEG-TRC105 were intravenously injected in 4T1 tumor-bearing mice. After 3 h p.i. (when 4T1 tumor uptake was at the peak based on PET imaging), mice were killed under anesthesia. Tumor, blood, and other important organs were collected for near-infrared optical imaging using IVIS spectrum in vivo imaging system (PerkinElmer, Waltham, MA). The signals from both QD (excitation/emission wavelength 605/700 nm) and DOX (excitation/emission wavelength 500/580 nm) were detected and analyzed.

### Histological Analysis

Two groups of three 4T1 tumor-bearing mice were each injected with FITC-QD@HMSN-PEG and FITC-QD@HMSN-PEG-TRC105 (10 pmol per mouse based on QD concentration) and euthanized at 3 h p.i. The 4T1 tumor, liver (positive control, which has high uptake of ^64^Cu-NOTA-QD@HMSN-PEG-TRC105 based on PET imaging), and muscle (negative control, which has low uptake of ^64^Cu-NOTA-QD@HMSN-PEG-TRC105 based on PET imaging) were frozen and cryo-sectioned for histological analysis. Frozen tissue slices with 6 μm thickness were fixed with cold acetone and stained for endothelial marker CD31, as described previously using a rat anti-mouse CD31 antibody and a Cy3-labeled donkey anti-rat IgG. All images from both FITC (representing the location of NPs in the tissues) and Cy3 (representing the location of vasculature) channels were acquired with a Nikon Eclipse Ti microscope.

## Results and Discussion

### Synthesis and Functionalization of QD@HMSN NPs

**Fig. 2:**
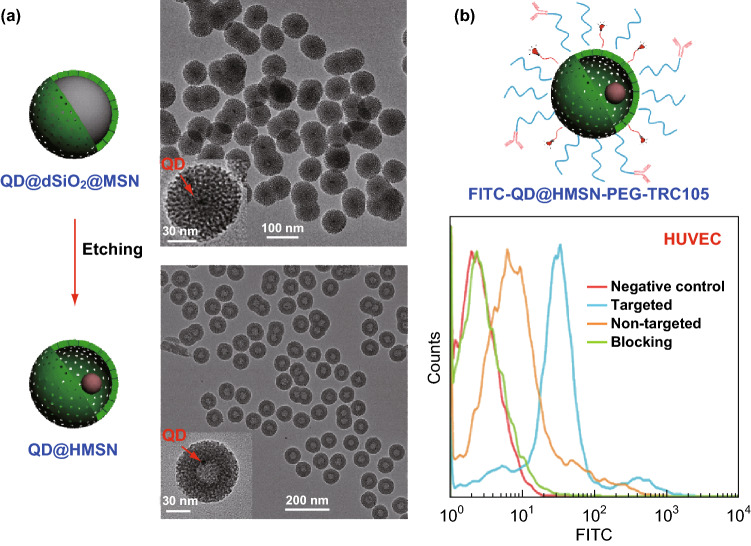
**a** The schemes and TEM images of QD@dSiO_2_@MSN and QD@HMSN NPs. **b** The scheme and flow cytometry analysis of QD@HMSN yolk/shell-structured silica nanosystem in HUVECs (CD105 positive)

The QD@HMSN NPs were synthesized by a two-step method, as shown in Fig. 1. The QD cores were first coated with dSiO_2_ through an oil-in-water reverse micro-emulsion silica coating approach [27]. This step effectively integrated hydrophobic QD cores into the silica nanosystem. By modifying the thickness of the dSiO_2_, we can easily control the size of the hollow cavity after etching. In the second step, as-prepared QD@dSiO_2_ NPs were further coated with MSN shell and then selectively etched out with Na_2_CO_3_ to generate an inner cavity. The final size of QD@HMSN was ~ 72 nm, which has a large cavity (~ 25 nm) and a QD core (~ 5 nm) inside each cavity, showing a distinctly different morphology compared to the QD@dSi_2_@MSN before etching (Fig. 2a). The surfactant CTAC was later removed via an extraction process by stirring the nanoparticles in NaCl: methanol solution (1 wt%) [28]. The silica pores (~ 2–3 nm) were exposed after the CTAC removal [29], which provide sufficient porous channels for efficient drug loading and release.

Amine groups (NH_2_) were introduced onto the surface of QD@HMSN by reacting with APTES in absolute ethanol for further functionalization [11]. PEG chains were conjugated onto amine-modified QD@HMSN to improve the solubility in physiological solutions and biocompatibility in vitro and in vivo. NOTA was employed as the coordination chelator for radiolabeling of ^64^Cu, an excellent radioisotope for PET with the half-life of 12.7 h. TRC105, a chimeric antibody that specifically binds to CD105, was conjugated for efficiently and specifically targeting CD105, which is exclusively expressed on the proliferating tumor vasculature. The hydrodynamic diameter of QD@HMSN was 76.3 ± 8.9 nm based on dynamic light scattering, whereas that of the final conjugate NOTA-QD@HMSN-PEG-TRC105 was increased to 98.1 ± 15.9 nm, suggesting successful conjugation of NOTA, PEG and TRC105 onto the surface of QD@HMSN.

### In Vitro CD105 Targeting

Fluorescent-dye FITC was conjugated onto QD@HMSN-PEG-TRC105 for in vitro angiogenesis targeting. As evidenced by flow cytometry results (Fig. 2b), significant enhancement was observed with the targeted group (FITC-QD@HMSN-PEG-TRC105), in comparison with the negative control (PBS), non-targeted group (FITC-QD@HMSN-PEG) and blocking group (FITC-QD@HMSN-PEG-TRC105 administrated after injection of a large dose of TRC105 antibodies). This result indicates the successful vasculature targeting and minimal nonspecific binding of QD@HMSN-PEG-TRC105 in cell culture.

### In Vivo Vasculature Targeting and PET Imaging

**Fig. 3:**
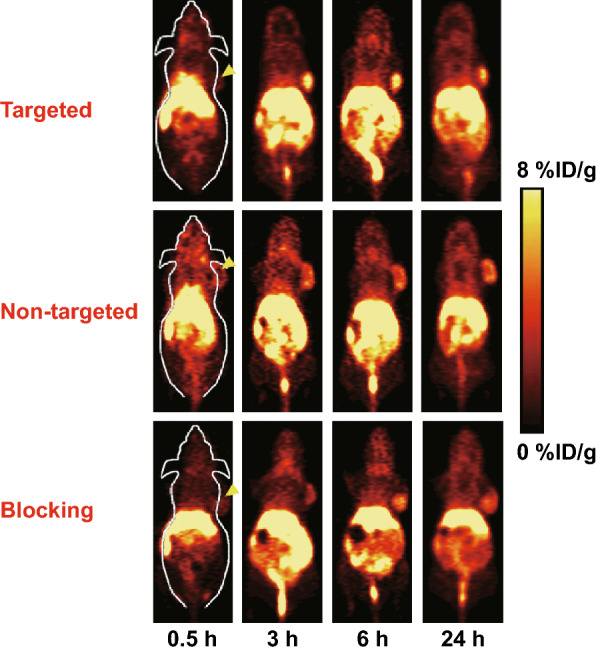
Serial coronal PET images of 4T1 tumor-bearing mice at different time points post-injection of ^64^Cu-NOTA-QD@HMSN-PEG-TRC105 (targeted group), ^64^Cu-NOTA-QD@HMSN-PEG (non-targeted group), or ^64^Cu-NOTA-QD@HMSN-PEG-TRC105 after a pre-injected blocking dose of TRC105 (blocking group). Tumors are indicated by arrowheads

**Fig. 4:**
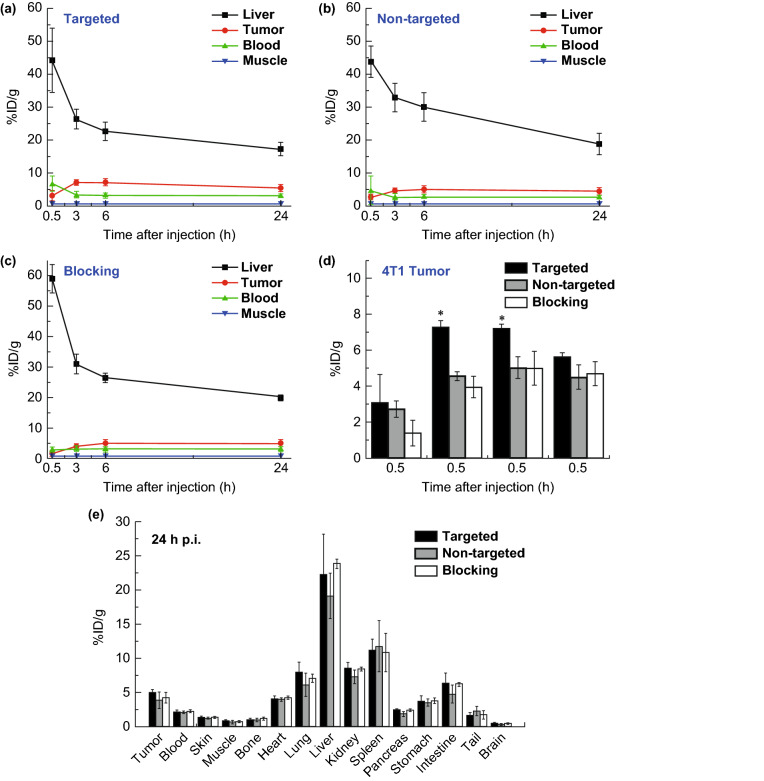
**a**–**c** Time–activity curves of the liver, 4T1 tumor, blood, and muscle upon intravenous injection of ^64^Cu-NOTA-QD@HMSN-PEG-TRC105 (targeted group), ^64^Cu-NOTA-QD@HMSN-PEG (non-targeted group), or ^64^Cu-NOTA-QD@HMSN-PEG-TRC105 after a pre-injected blocking dose of TRC105 (blocking group). **d** Comparison of the 4T1 tumor uptake in the three groups. **e** Biodistribution studies in 4T1 tumor-bearing mice at 24 h post-injection of the three groups of NPs. All data represent 4 mice per group (*P < 0.05)

^64^Cu was labeled onto NOTA-QD@HMSN-PEG-TRC105 (targeted group) and NOTA-QD@HMSN-PEG (non-targeted group) via simple mixing under mild conditions and purified with PD-10 desalting column. After radiolabeling, as-prepared ^64^Cu-NOTA-QD@HMSN-PEG-TRC105 (targeted group) and ^64^Cu-NOTA-QD@HMSN-PEG (non-targeted group) were intravenously injected into 4T1 tumor-bearing mice for in vivo vasculature targeting and PET imaging. The coronal PET images that contain the 4T1 tumors are shown in Fig. 3, and the quantitative data obtained from ROI analysis of the PET data are shown in Fig. 4.

The tumor uptake in the targeted group was prompt and persistent, manifesting as early as 3 h p.i. and remained visible after 24 h p.i. (3.1 ± 1.6, 7.2 ± 0.4, 7.2 ± 0.3, and 5.6 ± 0.3%ID/g at 0.5, 3, 6, and 24 h p.i., respectively; Fig. 4a). However, significantly lower tumor uptake was observed in non-targeted (2.7 ± 0.5, 4.6 ± 0.2, 5.0 ± 0.6, and 4.5 ± 0.7%ID/g at 0.5, 3, 6, and 24 h p.i., respectively; Fig. 4b) and blocking groups (1.4 ± 0.7, 3.9 ± 0.6, 4.9 ± 0.9, and 4.7 ± 0.7%ID/g at 0.5, 3, 6, and 24 h p.i., respectively; Fig. 4c). About 1.5-fold increase in tumor uptake was achieved with the targeted group than the non-targeted and blocking groups (*p* < 0.05 at 3 and 6 h p.i., respectively; Fig. 4d), suggesting the excellent targeting efficiency and specificity.

To the contrary, no significant increase from targeted group was found in other normal organs. For example, most of the NPs are eventually transported to the liver and cleared via hepatobiliary pathway [22]. In this study, slight reduction in liver uptake was observed in the targeted group (44.2 ± 9.8, 26.3 ± 3.0, 22.7 ± 2.8, and 17.2 ± 2.0%ID/g at 0.5, 3, 6, and 24 h p.i., respectively; Fig. 4a) than non-targeted (43.9 ± 4.8, 32.8 ± 4.4, 30.1 ± 4.4, and 18.8 ± 3.3%ID/g at 0.5, 3, 6, and 24 h p.i., respectively; Fig. 4b) and blocking group (58.8 ± 4.7, 31.0 ± 2.9, 26.6 ± 1.5, and 20.4 ± 0.3%ID/g at 0.5, 3, 6, and 24 h p.i., respectively; Fig. 4c), possibly because more NPs were trapped in tumor tissues in the targeted groups. In addition, the muscle which has no CD105 expression exhibited nearly identical uptakes from all three groups (targeted 0.8 ± 0.4, 1.0 ± 0.3, 0.9 ± 0.1, and 0.7 ± 0.1%ID/g; non-targeted 0.7 ± 0.1, 0.8 ± 0.1, 0.7 ± 0.1, and 0.7 ± 0.1%ID/g; blocking 0.4 ± 0.2, 0.7 ± 0.1, 0.7 ± 0.1, and 0.7 ± 0.1%ID/g at 0.5, 3, 6, and 24 h p.i., respectively), suggesting minimal nonspecific binding of ^64^Cu-NOTA-QD@HMSN-PEG-TRC105 to the normal organs. Furthermore, the blood uptakes from all three groups were also similar (targeted 6.7 ± 2.1, 3.3 ± 0.1, 3.3 ± 0.3, and 3.2 ± 0.4%ID/g; non-targeted 4.6 ± 4.4, 2.8 ± 0.7, 2.9 ± 0.3, and 2.8 ± 0.4%ID/g; blocking 2.9 ± 0.6, 2.9 ± 0.1, 3.2 ± 0.1, and 3.0 ± 0.2%ID/g at 0.5, 3, 6, and 24 h p.i., respectively), confirming the targeting specificity.

To further validate the accuracy of PET imaging, ex vivo biodistribution studies were conducted by wet weighting and measuring the radioactivities from tumor and other organs (Fig. 4e). The results corroborated well with the ROI analysis of PET images, where tumor uptake was significantly enhanced in the targeted group in comparison with the non-targeted and blocking groups.

### Drug Loading and Optical Imaging

**Fig. 5:**
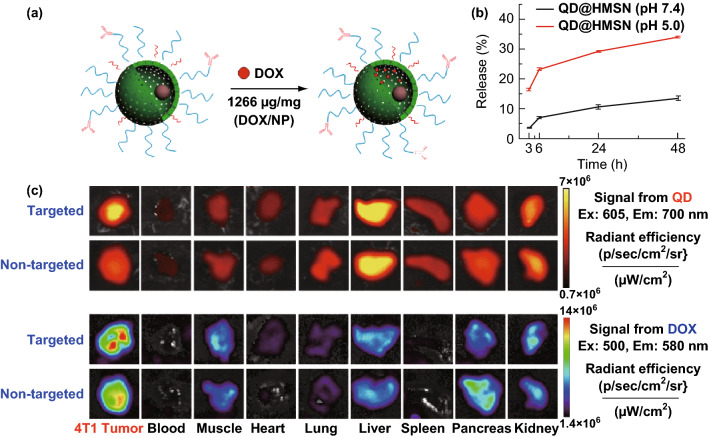
**a** A schematic illustration of DOX loading in QD@HMSN yolk/shell-structured silica nanosystem. **b** The drug release rates of QD@HMSN NPs at pH 7.4 and pH 5.0 at different incubation time points. **c** Fluorescence imaging of QD705 (*E*_*x*_: 605, *E*_*m*_: 700 nm) and DOX (*E*_*x*_: 500, *E*_m_: 580 nm) from both NOTA-QD@HMSN(DOX)-PEG-TRC105 (targeted group) and NOTA-QD@HMSN(DOX)-PEG (non-targeted group) in 4T1 tumor and other organs

One of the most important advantages of HMSN over MSN is the increased drug loading capacity. Typically, due to large surface area of MSN, the anticancer drug DOX can be easily loaded onto MSN via hydrophobic and electrostatic interactions, with a loading capacity of ~ 400–500 mg g^−1^ (DOX weight/NP weight) [29]. After introducing a large cavity inside the MSN shells, the loading capacity was remarkably increased to 1266 mg g^−1^ (DOX weight/NP weight) in as-designed yolk/shell QD@HMSN NPs (Fig. 5a). The increased drug loading can potentially benefit the efficacy of cancer chemotherapy [30–32] and also reduce the potential in vivo cytotoxicity from the nanocarriers since smaller dose of NPs will be needed for each treatment. In addition, the drug release rate is pH dependent (Fig. 5b). In normal physiological environment (pH 7.4), 13.5 ± 0.7% of DOX was released from QD@HMSN after 48-h incubation. However, when the pH decreased to ~ 5, drug release was dramatically accelerated and 34.0 ± 0.2% of DOX was released from QD@HMSN after 48-h incubation, due to decreased hydrophobic and electrostatic interactions between DOX and silica at lower pH values. Since tumors generally have lower pH values than normal tissues, as-prepared DOX-loaded QD@HMSN can be utilized as a promising pH-sensitive drug delivery system.

Although PET imaging is highly sensitive and quantitative, it can only represent the biodistribution of radioisotopes rather than the NPs [17–19], since the detachment of radioisotopes and chelators from NPs is inevitable when they circulate in the blood stream, accumulate in livers and other organs, and interact with proteins and other biological macromolecules. Therefore, another imaging modality is recommended to confirm the accuracy of PET imaging and depict the real biodistribution of NPs. In this study, QD705 (*E*_*x*_: 605, *E*_*m*_: 700 nm) inside each silica shell was employed for fluorescent imaging to understand the biodistribution of QD@HMSN NPs. In addition, anticancer drug DOX (*E*_*x*_: 500, *E*_*m*_: 580 nm) that was loaded in the cavity of QD@HMSN NPs was also imaged to further validate their biodistribution. As shown in Fig. 5c, the tumors exhibited significant differences between targeted (NOTA-QD@HMSN(DOX)-PEG-TRC105) and non-targeted groups (NOTA-QD@HMSN(DOX)-PEG) from QD- and DOX-based optical imaging. However, no difference was found between targeted and non-targeted groups in all the normal organs. Taken together, optical imaging of QD and DOX corroborated well with the PET imaging and confirmed the successful targeting of TRC105-conjugated QD@HMSN, which can serve as a multifunctional nanoplatform for dual-modality cancer diagnosis and drug delivery.

### Histological Analysis

**Fig. 6:**
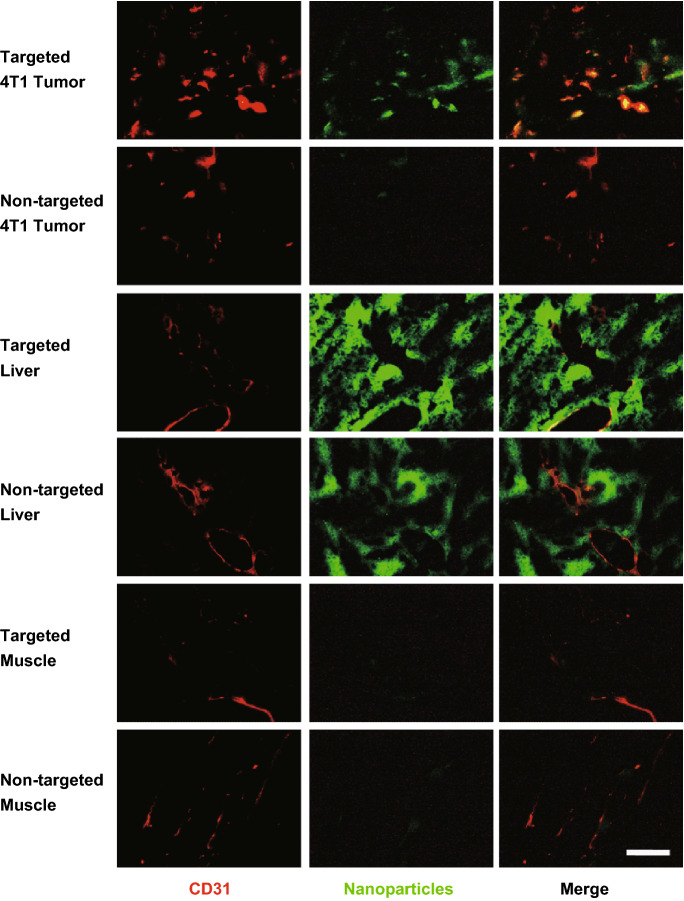
Histological analysis of various tissue slices for CD31 (red, with anti-mouse CD31 primary antibody) and NPs (green, FITC-conjugated QD@HMSN NPs) from both FITC-NOTA-QD@HMSN-PEG-TRC105 (targeted group) and FITC-NOTA-QD@HMSN-PEG (non-targeted group). Scale bar: 100 μm. (Color figure online)

Histological studies were conducted to evaluate the specificity of vasculature targeting. As shown in Fig. 6, excellent correlation was found between the signals from vasculature (CD 31, which is specifically expressed on vascular endothelial cells; red channel) and CD105-targeted NPs (FITC-QD@HMSN(DOX)-PEG-TRC105; green channel), indicating the excellent targeting specificity. Due to their relatively large size, minimal extravasation of NPs from the tumor vasculature was observed, which emphasizes the importance of vasculature targeting over tumor cell targeting. On the other hand, non-targeted group (FITC-QD@HMSN(DOX)-PEG) exhibited much lower tumor accumulation. Liver was selected as the positive control, since it is the major clearance organ of NPs. In our study, both targeted and non-targeted groups showed strong accumulation (green channel), and no correlation was found between the signals from vasculature and NPs in livers, suggesting that QD@HMSN NPs were captured by liver via nonspecific reticuloendothelial system (RES) uptake. In addition, minimal accumulation was found in muscle (negative control) in both targeted and non-targeted groups, which matched well with the results from PET imaging.

## Conclusion

Herein, we report a radiolabeled antibody-conjugated yolk/shell-structured silica nanosystem ^64^Cu-NOTA-QD@HMSN-PEG-TRC105. Significant boost of tumor uptake (1.5-fold increase at early time points) and excellent imaging contrast was achieved with specific tumor vasculature targeting. Compared to conventional MSN, the greatly enhanced drug loading capacity (1266 mg g^−1^, DOX/NP weight ratio) was achieved with yolk/shell QD@HMSN NPs, which can be used for improved chemotherapy of cancer. Taking advantage of both imaging techniques, PET/optical dual-modality imaging was utilized to cross-validate the imaging results, providing a more reliable imaging outcome. By combining vasculature targeting, pH-sensitive drug delivery and dual-modality imaging into a single platform, yolk/shell-structured silica nanosystem may be employed for the future image-guided tumor-targeted drug delivery, to further enhance the therapeutic efficacy and to enable cancer theranostics.
